# 528 Knowledge and Attitude of Health Care Staff in First Aid For Minor Burns

**DOI:** 10.1093/jbcr/irad045.125

**Published:** 2023-05-15

**Authors:** Ayse Gul Atay Doygaci, Ayse Ebru Abali, Elif Unlu, Santiago Santelis, Mehmet Haberal

## Abstract

**Introduction:**

Health care professionals are supposed to be well-versed for correct and timely first aid interventions in burns. This research was conducted to evaluate their level of knowledge and attitude for first aid in minor burns.

**Methods:**

Subjects were 402 volunteer health care staff in our university hospital. Study group involved nurses (n=163), medical technicians (n=94), technicians+patient-care support personnels (n=145). Data were collected by face-to-face interview technique using our "*Introductory Characteristics of the Participants*" and the "*Evaluation Form of the First Aid Knowledge Levels of the Health Professionals Regarding Minor Burns*" forms (p< 0.005).

**Results:**

Mean age was 29.7 yrs (minimum:20, maximum:54). Female to male ratio was 1:0,7. Of all subjects, nurses were 40.5%, 29.4% were medical technicians, 30.1% were technicians+patient-care staff. The rate of participants who stated that they have received first aid training on burns was 41.5%; but only 38.3% of the subjects stated that they could confidently provide first aid. Participants who correctly answered the form of *"First Aid Knowledge Levels of Health Professionals Evaluation Form for Minor Burns"* involved 76.9% of all subjects. Washing with water was preferred by 85.6% of the participants, 13.2% used additional methods following water (topical agents, remedies, ice, yogurt, egg white, cucumber, etc.), only 1.2% preferred methods other than water. High education level, receiving first aid training, and seniority in the profession and in the institution were the effective factors in providing the right first aid method (p< 0.005). Female (58.5%), single (59.8%) and child-free (61.7%) subject, and the subjects who have attended first aid education programs more recently were prone to provide correct first aid than the others (p< 0.005). However, knowledge about the factors affecting the severity of the burn injury was significantly high only among the subjects who received first aid training (p< 0.005).

**Conclusions:**

Many participants had basic knowledges on first aid for minor burns. However most did not have a comprehensive expertise and felt unconfident about providing first aid. Our results revealed that health care staff need education peculiar to them in addition to the basic awareness programs for burn injuries.

**Applicability of Research to Practice:**

First aid is the initial step of an appropriate burn care. Health care staff are supposed to be well-versed for correct and timely first aid interventions. Present definitive study provides basic information about knowledge level and attitudes of health care staff and it demonstrates useful data for specifying the future educational strategies.

